# The Neurogenesis Actuator and NR2B/NMDA Receptor Antagonist Ro25-6981 Consistently Improves Spatial Memory Retraining Via Brain Region-Specific Gene Expression

**DOI:** 10.1007/s12031-018-1083-5

**Published:** 2018-05-22

**Authors:** Marina A. Gruden, Alexander M. Ratmirov, Zinaida I. Storozheva, Olga A. Solovieva, Vladimir V. Sherstnev, Robert D. E. Sewell

**Affiliations:** 1 Federal State Budgetary Research Institution “Р. K. Anokhin Research Institute of Normal Physiology”, 125315, Baltiskaya St., 8, Moscow, Russia; 2 Serbsky State Scientific Center for Social and Forensic Psychiatry of Russian Ministry of Health, Kropotkinsky Per. 23, 19034 Moscow, Russia; 3 Cardiff School of Pharmacy and Pharmaceutical Sciences, Cardiff University, Redwood Building, King Edward VII Ave., Cathays Park, Cardiff, CF10 3NB UK

**Keywords:** Spatial memory, Water maze, NR2B/NMDA glutamate receptors, Ro25-6981, Neurogenesis, Gene expression

## Abstract

NR2B-containing NMDA (NR2B/NMDA) receptors are important in controlling neurogenesis and are involved in generating spatial memory. Ro25-6981 is a selective antagonist at these receptors and actuates neurogenesis and spatial memory. Inter-structural neuroanatomical profiles of gene expression regulating adult neurogenesis and neuroapoptosis require examination in the context of memory retrieval and reversal learning. The aim was to investigate spatial memory retrieval and reversal learning in relation to gene expression-linked neurogenetic processes following blockade of NR2B/NMDA receptors by Ro25-6981. Rats were trained in Morris water maze (MWM) platform location for 5 days. Ro25-6981 was administered (protocol days 6–7) followed by retraining (days 15–18 or 29–32). Platform location was tested (on days 19 or 33) then post-mortem brain tissue sampling (on days 20 or 34). The expression of three genes known to regulate cell proliferation (*S100a6*), differentiation (*Ascl1*), and apoptosis (*Casp-3*) were concomitantly evaluated in the hippocampus, prefrontal cortex, and cerebellum in relation to the MWM performance protocol. Following initial training, Ro25-6981 enhanced visuospatial memory retrieval performance during further retraining (protocol days 29–32) but did not influence visuospatial reversal learning (day 33). Hippocampal *Ascl1* and *Casp-3* expressions were correspondingly increased and decreased while cerebellar *S100a6* and *Casp-3* activities were decreased and increased respectively 27 days after Ro25-6981 treatment. Chronological analysis indicated a possible involvement of new mature neurons in the reconfiguration of memory processes. This was attended by behavioral/gene correlations which revealed direct links between spatial memory retrieval enhancement and modified gene activity induced by NR2B/NMDA receptor blockade and upregulation.

## Introduction

Recent evidence has implicated *N*-methyl-d-aspartate receptors (NMDARs) in several aspects of learning ability and behavioral flexibility in rodents (Delgado-García and Gruart 2017; Zhou and Wollmuth 2017)*.* In addition, it has been demonstrated that NMDARs are important not only for the acquisition of new memories but also for the decay of memories acquired previously (Shinohara and Hata 2018). There is also ample evidence that neurogenesis (proliferation, differentiation, and migration of both neuronal and glial cells) occurs in the adult brain, notably in anatomical structures which are associated with learning and memory (Toda and Gage 2018; Ming and Song 2011; Nicola et al. 2015: Opendak and Gould 2015).

Data concerning the plasticity and potential of adult neural stem and progenitor cells has accumulated over the years. It is known that alongside astrocyte gene expression (Lisachev et al. 2010), these cells can give rise not only to neurons but also to astrocytes, reactive astrocytes, and ultimately to oligodendrocytes through genetic manipulation (Encinas and Fitzsimons 2017). In this connection, newborn neurons are integrated in neuronal pre-existing nets and underlie new skills whereby their survival, apoptotic death, and extent of maturation are contingent upon the overall environmental and metabolic conditions (Kee et al. 2007; Kempermann 2012; Encinas et al. 2013). Moreover, the involvement of new neurons in learning and memory mechanisms is also determined by their degree of maturity (Deng et al. 2010; Richetin et al. 2015). The excitatory amino-acid glutamate is known to regulate adult neurogenesis in the hippocampus. This neurotransmitter operates through NMDA receptors, which modulate not only the proliferation of progenitor cells but also the rate of neurogenesis in the dentate gyrus (Nácher and McEwen 2006; Nácher et al. 2007; Thakurela et al. 2015). Consequently, stimulation of NMDA receptors reduces neurogenesis while NMDA antagonism has the reverse effect (Cameron et al. 1995). However, the mechanisms by which these processes influence memory formation are not well established.

One experimental approach for studying the role of newborn cells in integrative brain activity involves stimulation or blockade of neurogenetic stages. NMDA blockade in particular promotes neuronal propagation in the hippocampus (Cameron et al. 1995; Okuyama et al. 2004) both in the young as well as the aged state (Nácher et al. 2003; Nácher and McEwen 2006). The physiological actions of NMDA receptors largely depend on the constituent NR2 subunits, and in the hippocampus, NR2A/B subunits by themselves exemplify macromolecular complexes with NR1 (Kiselycznyk et al. 2015). The NR2B subunit is prevalent in human and animal neural stem cells, and it has been postulated that NMDA receptors comprising this subunit may be essential in controlling neuronal propagation and memory (Hu et al. 2008; Nakazawa et al. 2004). In the late 1980s, a new class of NMDA antagonists, typified by the phenylethanolamine ifenprodil, was identified (Chenard and Menniti 1999). In this regard, ensuing pharmacological manipulation with selective antagonists at NMDA receptors incorporating the NR2B subunit (NR2B/NMDA receptors) such as the potent, selective, and activity-dependent antagonist of NR2B/NMDA receptors, namely Ro25-698, has been studied (Fischer et al. 1997; Lynch et al. 2001. Szczurowska and Mareš 2015). In animals, the NR2B subunit is expressed in cell precursors which are localized in different brain areas and differentiated into granular neurons in the hippocampus (Nácher et al. 2007). It has been shown that in mice, Ro25-6981 instigates an increase in the quantity of newly born BrdU-labeled cells at the 29–34-day-old stage in the rat hippocampal dentate gyrus. This result correlated with facilitated expression of visuospatial skill in the Morris water maze (MWM) (Hu et al. 2008). However, it was also discovered that there was an increase in 1–6-day-old novel cells which did not influence learning and memory processes (Hu et al. 2008). Along with the functional role of different aged cells developed in post-natal ontogenesis, considerable interest has been focused on the learning ability of animals and the degree of neurogenesis (Storozheva et al. 2015). It has been reported that in individuals with a high capacity for learning, greater numbers of newborn cells are generated and survive in comparison with individuals displaying lower learning capacities (Coras et al. 2010). In earlier studies, it has also been disclosed that Ro25-6981 in a dose which is known to stimulate neurogenesis (Hu et al. 2008) did not influence repeated and reversal learning when administered directly before repeated training (Soloviova et al. 2012). However, Ro25-6981 facilitated the formation of spatial skill in those animals with initial inferior learning ability (Soloviova et al. 2011). More recent data has suggested that spatial reversal learning is sensitive to Ro25-6981 whereby NR2B/NMDA receptor signaling may be implicated in behavioral plasticity involved in updating spatial information (Clark et al. 2017).

Genetic regulation of short- and long-term memory is a topical area of research. However, inter-structural neuroanatomical profiles of gene expression regulating adult neurogenesis and neuroapoptosis require special consideration in the context of memory. The expression of three genes known to regulate cell proliferation (*S100a6*), differentiation (*Ascl1*), and apoptosis (*Casp-3*) have been concomitantly evaluated in the rat hippocampus, prefrontal cortex, and cerebellum with respect to water maze spatial memory performance. The gene expression outcome patterns supported the concept of a hippocampal involvement in the acquisition of memory in addition to a complementary connection between the prefrontal cortex and cerebellum (Gruden et al. 2013)

In light of this, there are no data concerning NR2B/NMDA receptor antagonism on gene expression implicated in neurogenesis and neuroapoptosis in different brain structures either in naïve animals or during consolidation and reconsolidation of spatial performance. Therefore, the aim was to study spatial memory retrieval, reversal learning, and gene expression following blockade of NR2B/NMDA receptors by the neurogenesis actuator Ro25-6981 as a potential memory enhancer. Regarding this point, hippocampal, prefrontal cortical and cerebellar brain areas were specifically selected for study. This was because it has been previously shown that there are integrative relationships between *Casp3*, *Ascl1*, and *S100a6* genes in all three of these neuroanatomical structures with respect to spatial memory (Gruden et al. 2013)

## Experimental Procedures

All experimental procedures were carried out in accordance with the National Institute of Health Guide for the Care and Use of Laboratory Animals (NIH publication No 80-23, revised 1996). They also conformed to the UK Animal Scientific Procedures Act, as well as the European Community Council Directive (86/609/EEC). They were additionally approved by the Animal Care and Use Committee of the Federal State Budgetry Research P. K. Anokhin Research Institute of Normal Physiology.

Ro25-6981 (α*R*,β*S*)-α-(4-hydroxyphenyl)-β-methyl-4-(phenylmethyl)-1-piperidinepropanol maleate was supplied by Sigma-Aldrich, USA.

### Animals

Age-matched male Wistar rats weighing 230 ± 20 g (*n* = 98) were randomly allocated to the following groups:

Group 1: MWM training animals that underwent water maze training without any treatment; group 2: active controls that were non-injected untrained animals allowed to act as swim controls; group 3: training animals 14 days post-Ro25-6981 dosing; group 4: training animals 14 days post-saline vehicle injection; group 5: training animals 28 days post-Ro25-6981 administration; group 6: training animals 28 days post-saline vehicle injection; group 7: naïve rats killed 14 days after Ro25-6981 administration plus post-mortem brain tissue sampling; group 8: naïve rats killed 14 days after saline vehicle administration plus post-mortem brain tissue sampling; group 9: naïve rats killed 28 days after Ro25-6981 administration plus post-mortem brain tissue sampling; group 10: naïve rats killed 28 days after saline vehicle administration plus post-mortem brain tissue sampling. Each group consisted of a minimum of 10 up to a maximum of 14 animals.

### Drug Administration

**Fig. 1:**
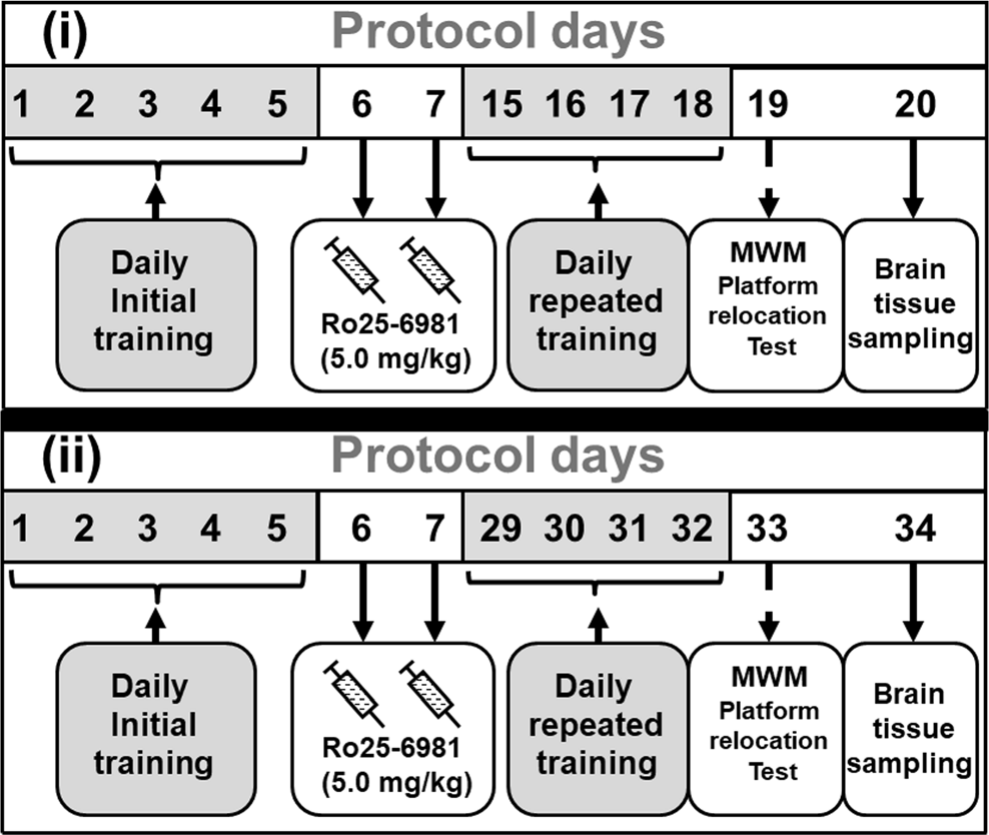
Scheme showing the experimental protocols. Protocol (i). On days 1–5, rats underwent daily initial training in the Morris water maze (MWM) followed on days 6 and 7 by Ro25-6981 (5.0 mg/kg i.p.) administration, then daily repeated training on days 15–18. On day 19, the animals were exposed to a platform relocation test (reversal learning). Lastly, on day 20, animals were killed and post-mortem regional brain samples were obtained. Protocol (ii). On days 1–5, rats underwent daily initial training in the Morris water maze (MWM) followed on days 6 and 7 by Ro25–6981 (5.0 mg/kg i.p.) administration, then daily repeated training on days 29–32. On day 33, the animals were exposed to a platform relocation test (reversal learning). Lastly, on day 34, animals were killed and post-mortem regional brain samples were obtained

In animals from experimental groups 3, 5, 7, and 9, Ro25-6981 (5.0 mg/kg) dissolved in saline vehicle was administered intraperitoneally (i.p.) (Hu et al. 2008; Duffy et al. 2008). All control groups were administered an identical volume of saline vehicle (1.0 ml/kg). Injections were performed twice, the first being given 24 h after the last session of initial training and the second 24 h later (i.e., protocol days 6 and 7 in Fig. 1).

### Water Maze Paradigm

The water maze was comprised of a gray pool (circular in shape and height 60 cm × 80 cm radius) marked into four quadrants containing water (22.0 ± 1.0 °C) to a 40-cm depth and rendered opaque by the addition of a small quantity of powdered milk, and there were external cues from the room (Gruden et al. 2013; Sewell et al. 2005). A clear Perspex escape platform (radius = 12 cm) was located in the center of one quadrant at a 2.0-cm depth. Assessment of task performance in the MWM was carried out with the aid of video monitoring, and initial training was accomplished over protocol days 1–5 (Fig. 1).

In each trial, the time taken to escape onto the hidden platform (swimming latency, s) within 60 s was recorded and followed by a 15-s occupation period on the platform, the intertrial interval being 60 s.

Each training session consisted of five successive trials, there being an intertrial period of 60 s with the platform position in the center of the quadrant, and individual trials involved random placement in a quadrant devoid of the platform. Escape latency onto the platform within a period of 60 s was logged then followed by a rest period before the next trial. Animals which did not find the platform were gently directed to the platform and allowed a 15-s occupancy time before resting. The active control group was encouraged to swim with the platform removed over the 5-day training period.

### Training, Dosing, Testing, and Tissue Sampling Protocols

#### Protocol (i)

Initial water maze training was performed daily for the first 5 days, then on days 6 and 7, animals were administered either saline vehicle or Ro25-6981 (5.0 mg/kg) intraperitoneally (Soloviova et al. 2011, 2012) followed by a modified behavioral protocol. Henceforth, daily repeated training was implemented on days 15–18 inclusively followed on day 19 by a platform relocation test (reversal learning) and later tissue sampling was performed on day 20 from post-mortem brains (Fig. 1).

#### Protocol (ii)

Initial water maze training was carried out daily for the first 5 days, then on days 6 and 7, animals were administered either saline vehicle or Ro25-6981 (5.0 mg/kg) intraperitoneally (Soloviova et al. 2011, 2012) followed by a modified behavioral protocol. Thus, daily repeated training was implemented on days 29–32 inclusively followed on day 33 by a platform relocation test (reversal learning) and subsequent tissue sampling on day 34 from post-mortem brains (Fig. 1).

#### Real-Time PCR Analysis

The day subsequent to the last platform relocation reversal learning water maze test (i.e., the 20th day in protocol (i) or the 34th day in protocol (ii)), animals were killed and brain structures dissected on ice. Using our published procedure, RNA from brain structures was isolated and purified from a genome DNA admixture by DNase 1 treatment and the RNA concentration determined fluorometrically by means of a Qubit RNA Assay Kit (Invitrogen, USA). Reverse transcription was then accomplished using RNA (50 ng), revertase M-MLV (200 U), and oligo-(dT) 15 plus RNAse inhibitor at 37 °C for 105 min, the resulting DNA being diluted (×10) then freeze stored. Real-time PCR was performed repetitively on samples containing diluted cDNA (1.0 μl), ready primer mixture (0.5 μl), qPCRmix-HS SYBR (5.0 μl), qPCRmix-HS SYBR (5.0 μl), and deionized water made up to 25.0 μl according to our previously described protocol (Gruden et al. 2013). Evaluation of gene expression levels were executed using the β-actin gene as a reference (Joo et al. 2008) and calculated according to Livak and Schmittgen (2001).

#### Statistics

Repeated-measures ANOVA was used for behavioral results analysis and the Mann-Whitney U test utilized to verify statistical differences in mRNA expression. Spearman’s rank coefficient (rS) computation was employed to identify any possible correlations among brain structure mRNA expressions.

## Results

### Behavior

#### Performance in Daily Initial Pre-Drug Training, Then Repeated Training and a Reversal Learning Test in the Morris Water Maze after Ro25-6981 Treatment

**Fig. 2:**
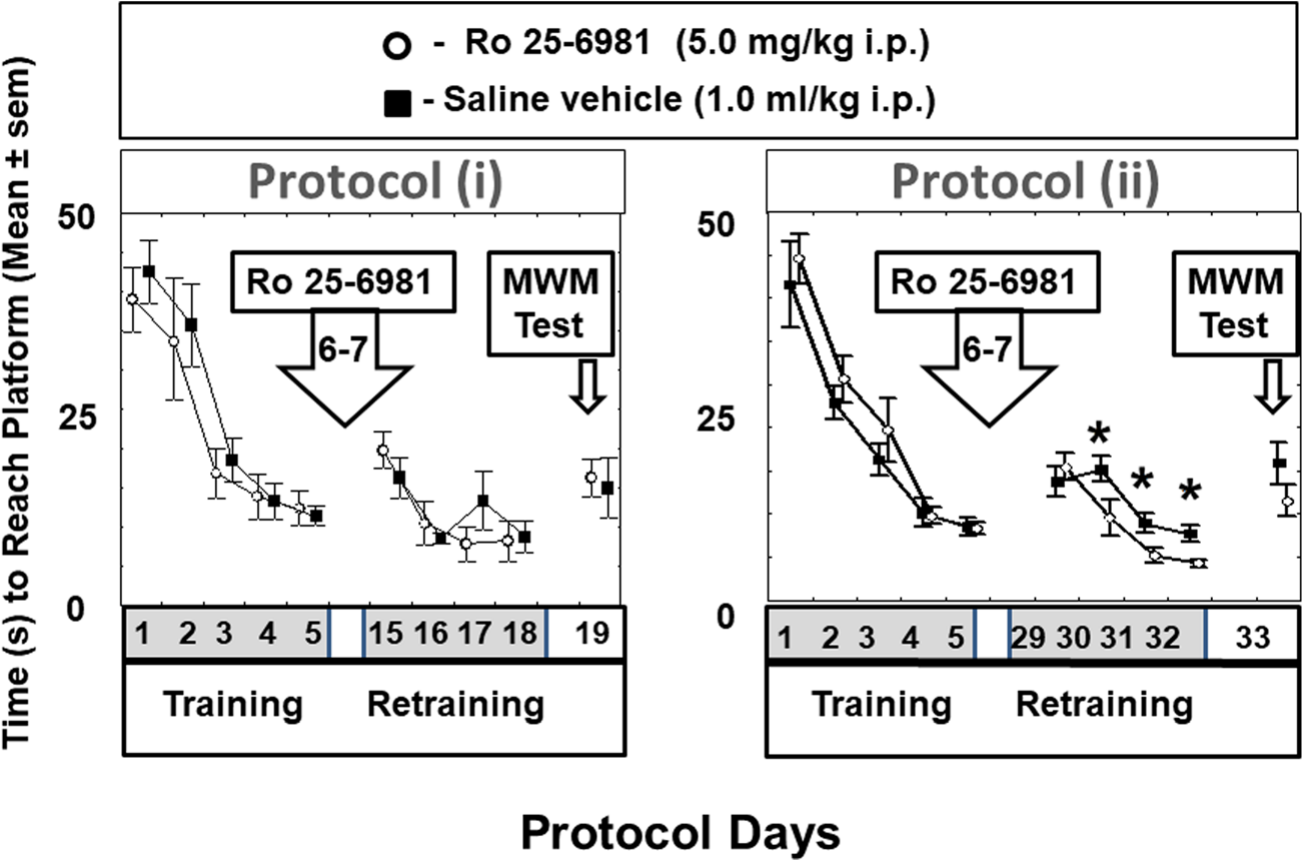
Latency to reach the platform for rats in the water maze (MWM) during initial training and after Ro25-6981 administration in retraining (further training) and the reversal learning procedure (RLP) in protocol (i) and (ii). Protocol (i). Mean MWM platform latencies (s) are shown for training days 1–5 then Ro25-6981 (5.0 mg/kg i.p.) or saline vehicle (1.0 ml/kg i.p.) administration on days 6 and 7 followed by retraining on days 15–18 and the MWM platform relocation test (MWM PRT, i.e., reversal learning) on day 19. Protocol (ii). Mean MWM platform latencies (s) are shown for training days 1–5 then Ro25-6981 (5.0 mg/kg i.p.) or saline vehicle (1.0 ml/kg i.p.) administration on days 6 and 7 followed by retraining on days 29–32 and the MWM platform relocation test (MWM PRT, i.e., reversal learning) on day 33. *P < 0.05 compared to saline vehicle-treated control animals

In the initial 5-day phase of training in protocol (i), there were no significant differences in platform latencies among control or experimental groups at any time before drug administration (Fig. 2). In the initial 5-day phase of the training protocol: (i) there was a significant effect of training day [F(4, 87) = 25.06, *P* < 0.001] but no effect of group [F(2.21) = 0.06, *P* > 0.1] or group × trial interaction [F(4, 87) = 0.49, *P* > 0.1] was observed.

After Ro25-6981 treatment, in the first 2 days of repeated learning (repeated training days 15 and 16 in Fig. 2), animals in both experimental groups were not significantly different in their platform latencies. Additionally, on repeated training days 17 and 18, the platform latencies had returned to the day 5 pre-drug levels. Repeated-measures one-way ANOVA revealed a significant effect of the retraining day [F(3.65) = 13.07, *P* < 0.001] but no effect of group [F(2.21) = 0.02, *P* > 0.1] or group × trial interaction [F(4.87) = 0.61, *P* > 0.1] was observed.

**Fig. 3:**
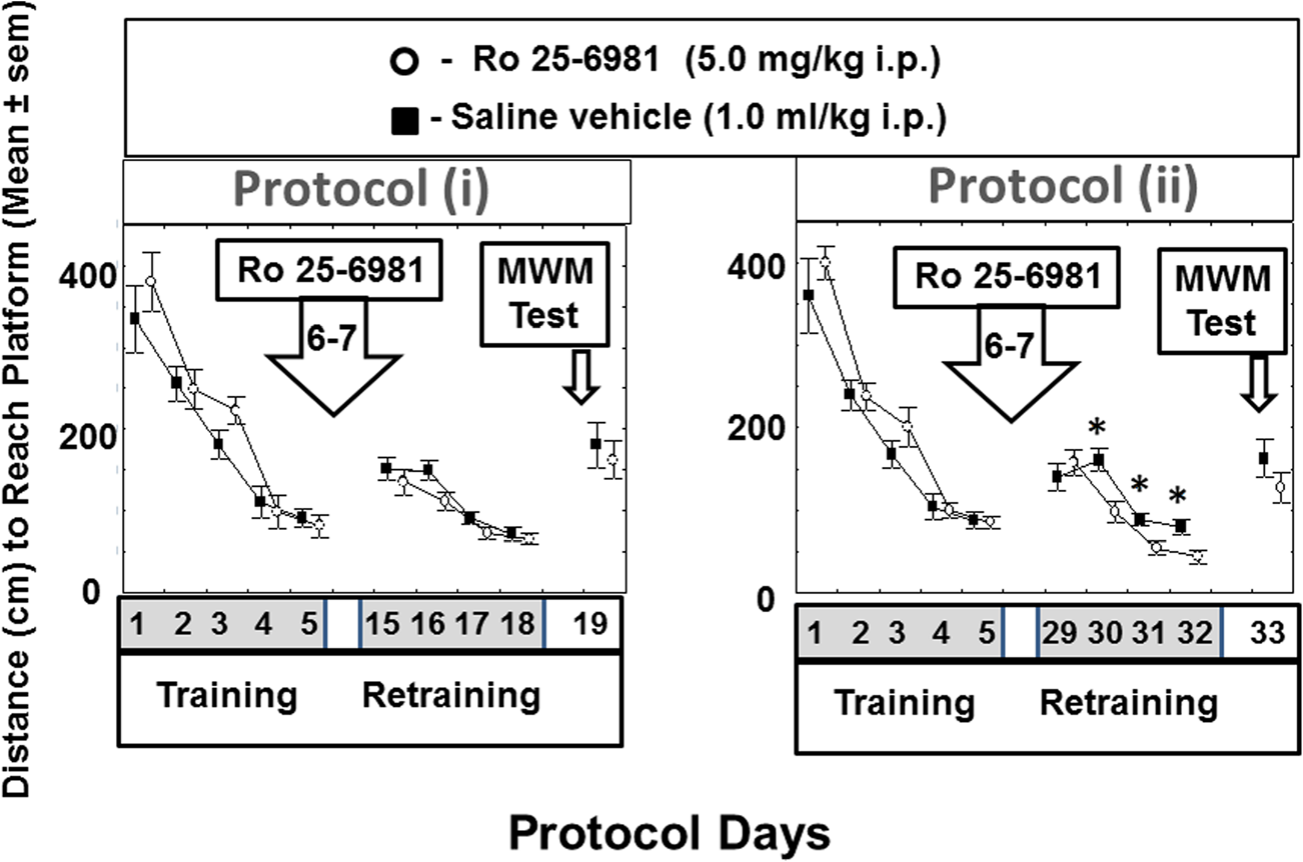
Distance to reach the platform for rats in the water maze (MWM) during initial training and after Ro25-6981 administration in retraining (further training) and the reversal learning procedure (RLP) in protocol (i) and (ii). Protocol (i). Mean distance (cm) to reach MWM platform are shown for training days 1–5 then Ro25-6981 (5.0 mg/kg i.p.) or saline vehicle (1.0 ml/kg i.p.) administration on days 6 and 7 followed by retraining on days 15–18 and the MWM platform relocation test (MWM PRT, i.e., reversal learning) on day 19. Protocol (ii). Mean distance (cm) to reach MWM platform location times (s) are shown for training days 1–5 then Ro25-6981 (5.0 mg/kg i.p.) or saline vehicle (1.0 ml/kg i.p.) administration on days 6 and 7 followed by retraining on days 29–32 and the MWM platform relocation test (MWM PRT, i.e., reversal learning) on day 33. *P < 0.05 compared to saline vehicle-treated control animals

What is more, throughout the whole protocol, there were no differences between the platform location times expressed in animals after Ro25-6981 administration compared with saline controls and this absence of difference persisted even in the platform relocation test (reversal learning) [F(2.21) = 0.5, *P* > 0.1] (i.e., day 19, Fig. 2). Similar results were obtained when the distance to reach platform was analyzed (Fig. 3).

In protocol (ii) similarly, there were no differences in platform location times between animal groups in the first 5 days of initial training. The effect of training day was highly significant [F(4.88) = 34.52, *P* < 0.001], but no effect of group [F(2.22) = 1.72, *P* > 0.1] or group × day interaction [F(4.88) = 1.15, *P* > 0.1] was observed.

After Ro25-6981 treatment, on the first 2 days of repeated learning (repeated training days 15 and 16 in Fig. 2), animals in both experimental groups were not significantly different in their platform latencies [F(2.22) = 1.89, *P* > 0.1]. However, repeated-measures one-way ANOVA revealed a significant effect of group [F(4.88) = 3.78, *P* < 0.05], day [F(3.66) = 23.45, *p* < 0.001], and group × day interaction [F(4.88) = 4.39, *P* < 0.01].

Post hoc analysis revealed that the saline and drug-treated group latencies then began to diverge on day 30 and this consequent Ro25-6981-induced differential attained statistical significance (*P* < 0.05) on days 30, 31, and 32 of the protocol though there was no disparity detected in the platform relocation test (reversal learning) (i.e., day 33, Fig. 2). [F(1.22) = 1.04, *P* > 0.1].

Similar results were obtained when the distance to reach the platform was analyzed (Fig. 3).

### Gene Expression

#### *Ascl1*, *S100a6*, and *Casp3* Gene Expression Analysis

In molecular genetic experiments, we have previously reported brain regional specificities in *Ascl1*, *S100a6*, and *Casp3* gene expression within the prefrontal cortex, hippocampus, and cerebellum (Gruden et al. 2013). In the current study, gene expression was examined in the same brain areas 14 and 28 days after Ro25-6981 (5.0 mg/kg i.p.) administration in parallel with initial pre-treatment training, post-treatment repeated training followed on day 19 or 33 by a platform relocation test (reversal learning) in the Morris water maze.

#### *Ascl1* Expression 14 and 28 Days after Ro25-6981 Double Treatment Commencing on Protocol Day 6

**Fig. 4:**
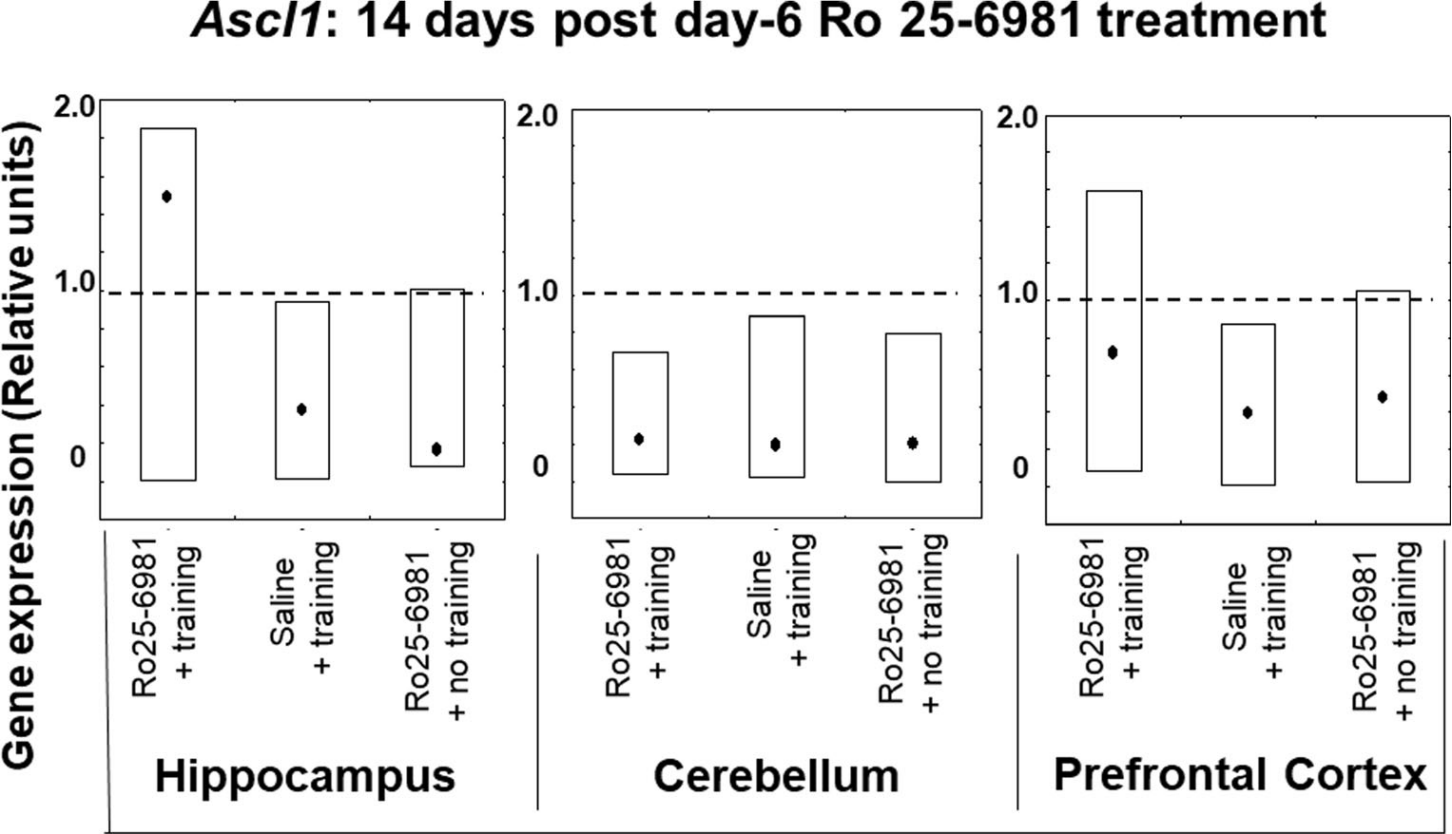
*Ascl1* gene expression in rat hippocampus, cerebellum, and prefrontal cortex 14 days after 2-day treatment with Ro25-6981 (5.0 mg/kg i.p.) with or without training compared to controls with or without training. *Ascl1* gene expression (relative units) in saline non-trained control animal brain structures (dotted lines). Points shown are medians with boxes displaying upper and lower interquartile range values

**Fig. 5:**
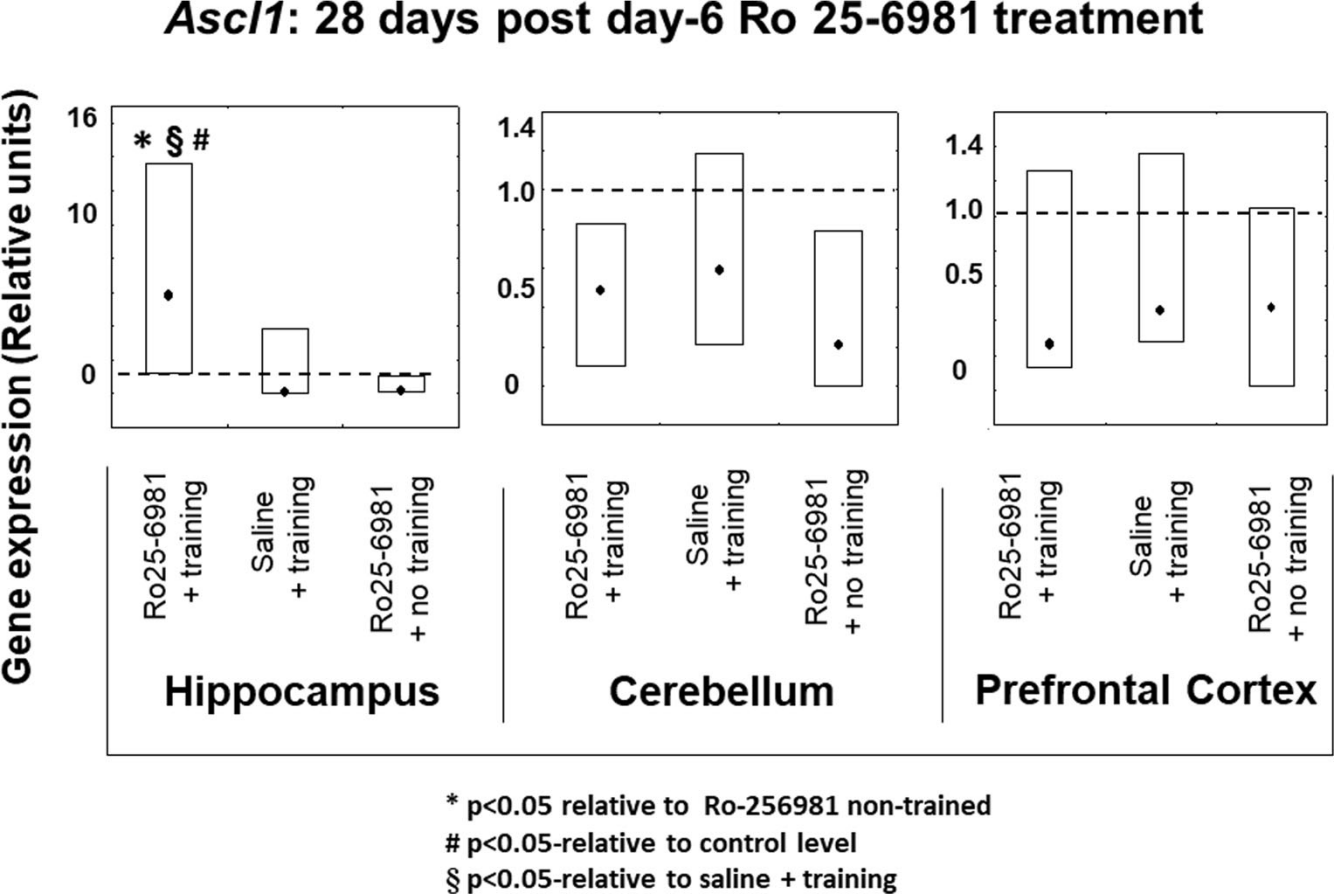
*Ascl1* gene expression in rat hippocampus, cerebellum, and prefrontal cortex 28 days after 2-day treatment with Ro25-6981 (5.0 mg/kg i.p.) with or without training compared to controls with or without training. *Ascl1* gene expression (relative units) in saline non-trained control animal brain structures (dotted lines). Points shown are medians with boxes displaying upper and lower interquartile range values. *P < 0.05 versus Ro25-6981 + no training, #*P* < 0.05 versus control (dotted line), §*P* < 0.05 versus saline + training

Examination of *Ascl1* transcription in the three brain areas disclosed that expression levels were not changed 14 days after protocol day 6 and 7 treatments in groups receiving Ro25-6981 or saline plus training and Ro25-6981 without training in comparison with the controls (Fig. 4). However, 28 days after treatment, hippocampal *Ascl1* expression levels only were elevated (*P* < 0.05) in those animals receiving Ro25-6981 plus training compared to those administered Ro25-6981 or the controls without training and saline plus training. In the case of the prefrontal cortex and cerebellum, there were no differences in expression levels of *Ascl1* between all groups and compared to controls 28 days following treatment (Fig. 5).

#### *S100a6* Expression 14 and 28 Days after Ro25-6981 Double Treatment Commencing on Protocol Day 6

**Fig. 6:**
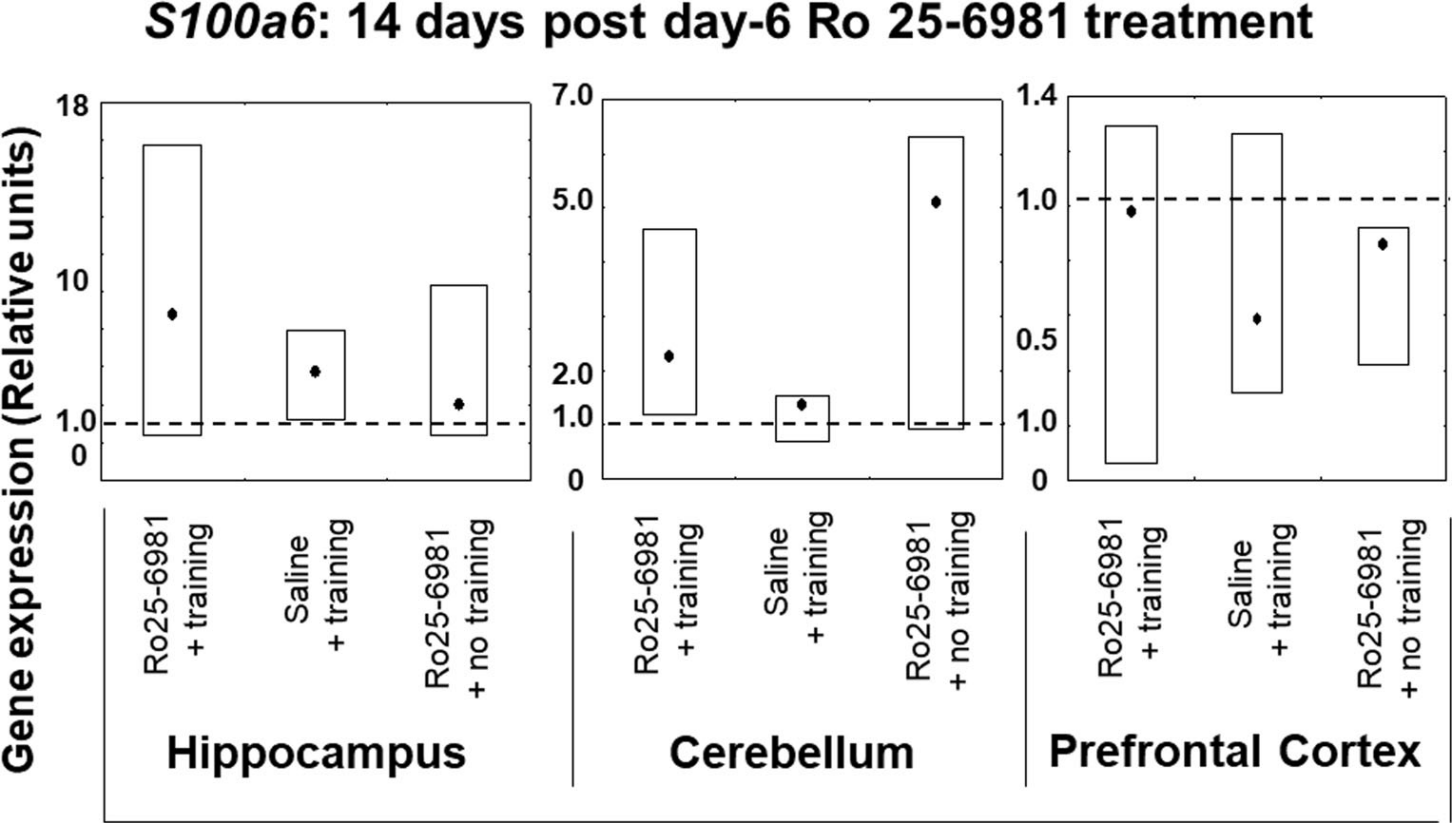
*S100a6* gene expression in rat hippocampus, cerebellum, and prefrontal cortex 14 days after 2-day treatment with Ro25-6981 (5.0 mg/kg i.p.) with or without training compared to controls with or without training. *S100a6* gene expression (relative units) in saline non-trained control animal brain structures (dotted lines). Points shown are medians with boxes displaying upper and lower interquartile range values

**Fig. 7:**
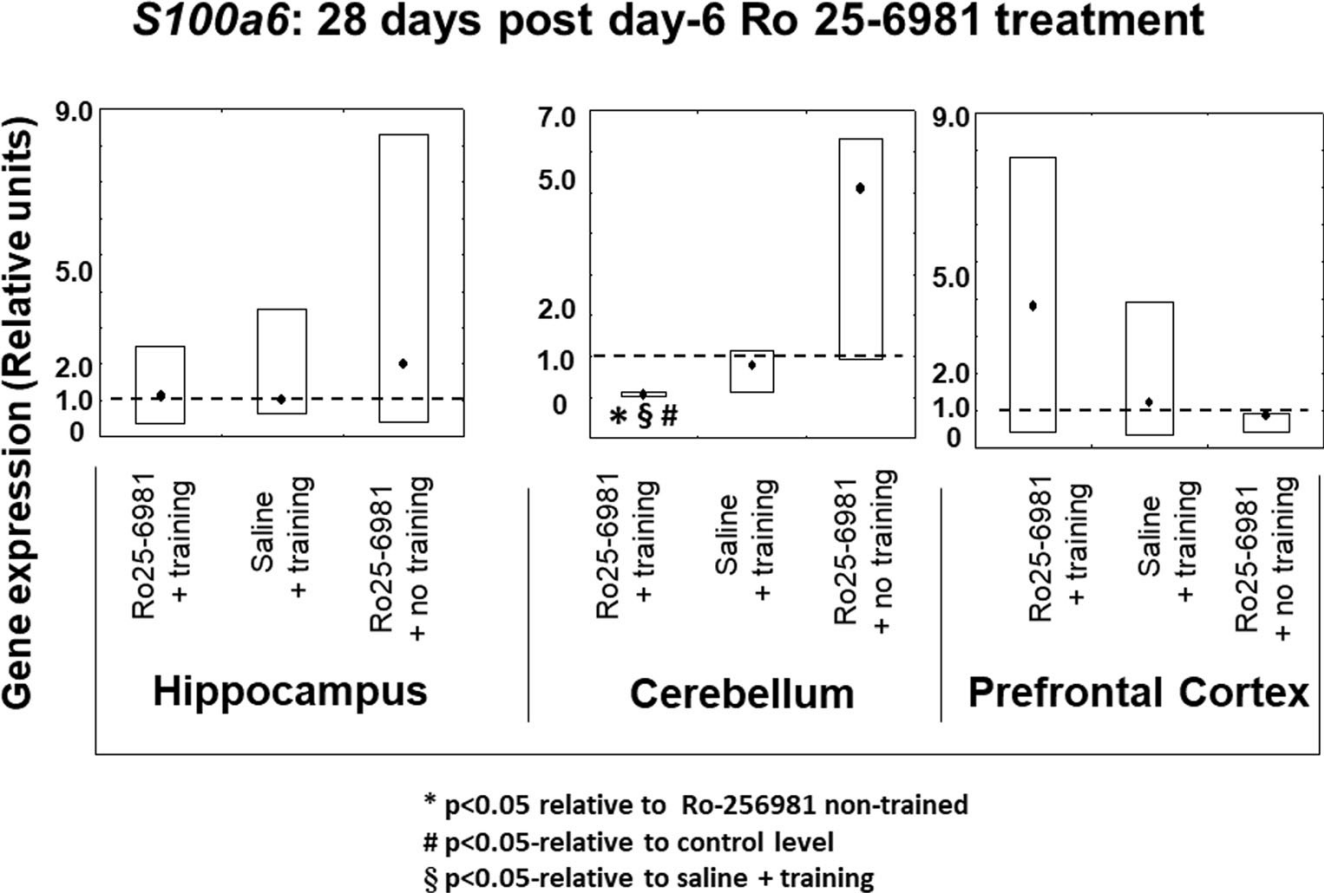
*S100a6* gene expression in rat hippocampus, cerebellum, and prefrontal cortex 28 days after 2-day treatment with Ro25-6981 (5.0 mg/kg i.p.) with or without training compared to controls with or without training. *S100a6* gene expression (relative units) in saline non-trained control animal brain structures (dotted lines). Points shown are medians with boxes displaying upper and lower interquartile range values. *P < 0.05 versus Ro25-6981 + no training, #*P* < 0.05 versus control (dotted line), §*P* < 0.05 versus saline + training

Fourteen days after double-drug treatment starting on protocol day 6, there were no differences in *S100a6* gene expression between any of the animal groups and also compared to the controls in the three brain structures under study (Fig. 6). This absence of modified gene expression with respect to brain structure differed from the pattern observed 28 days after drug treatment where the expression of *S100a6* was considerably diminished (*P* < 0.05) in comparison with the other groups as well as the controls exclusively in the cerebellum (Fig. 7). It was also noted that there were no significant changes in hippocampal or prefrontal cortical *S100a6* expression observed in any of the treated groups (Fig. 7).

#### *Casp-3* Expression 14 and 28 Days after Ro25-6981 Double Treatment Commencing on Protocol Day-6

**Fig. 8:**
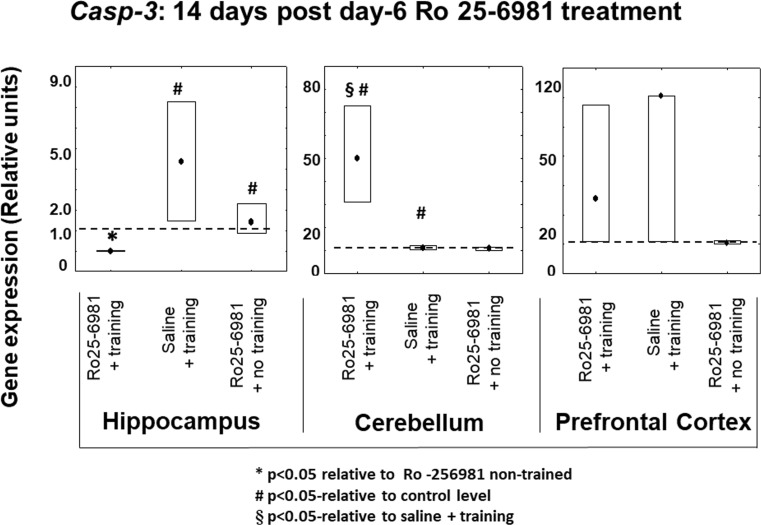
*Casp-3* gene expression in rat hippocampus, cerebellum, and prefrontal cortex 14 days after 2-day treatment with Ro25-6981 (5.0 mg/kg i.p.) with or without training compared to controls with or without training. *Casp-3* gene expression (relative units) in saline non-trained control animal brain structures (dotted lines). Points shown are medians with boxes displaying upper and lower interquartile range values. *P < 0.05 versus Ro25-6981 + no training, #*P* < 0.05 versus control (dotted line), §*P* < 0.05 versus saline + training

**Fig. 9:**
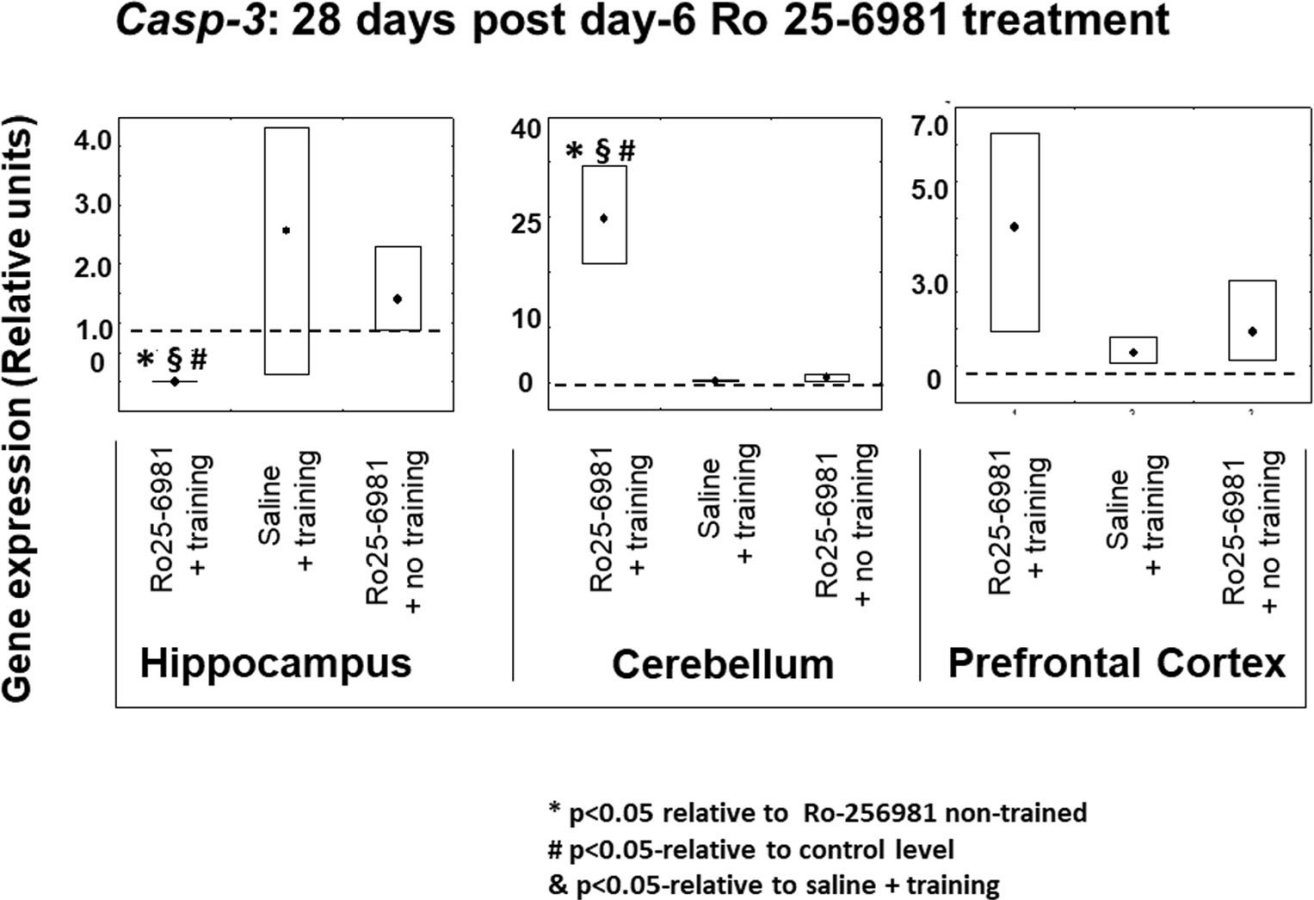
*Casp-3* gene expression in rat hippocampus, cerebellum, and prefrontal cortex 28 days after 2-day treatment with Ro25-6981 (5.0 mg/kg i.p.) with or without training compared to controls with or without training. *Casp-3* gene expression (relative units) in saline non-trained control animal brain structures (dotted lines). Points shown are medians with boxes displaying upper and lower interquartile range values. *P < 0.05 versus Ro25-6981 + no training, #*P* < 0.05 versus control (dotted line), §*P* < 0.05 versus saline + training

In the case of *Casp-3* transcription 14 days after double-drug treatment (protocol days 6 and 7), in the hippocampus, there was a marked inhibition of its expression in the animals which received Ro25-6981 along with training compared to those given Ro25-6981 without any training. In addition, there was an increase in *Casp-3* expression in subjects which underwent training coupled with saline administration versus the controls. On the other hand, in the cerebellum, there was a contrasting profile because the expression of *Casp-3* in the group given Ro25-6981 plus training, rather than being reduced, it was significantly elevated (*P* < 0.05) in comparison not only with controls but also with those individuals given saline plus training and Ro25-6981 without training (Fig. 8). There was an almost identical pattern of outcomes 28 days after double-drug treatment (Fig. 9) in the hippocampus, and *Casp-3* expression was reduced in the group treated with Ro25-6981 plus training compared to saline with training as well as the controls. Analogous to the 14-day results in the cerebellum, at 28 days after dual treatment, there was a marked increase in cerebellar *Casp-3* transcription but no change in the prefrontal cortex.

#### Correlation Analyses between Gene Expression and Behavioral Swimming Intervals

Protocol day independent correlation analyses between gene expression and behavioral swimming intervals after drug treatment were performed. Thus, there was a negative correlation between the unmodified prefrontal cortical *Ascl1* expression and overall repeated training swimming time (*r*_*s*_ = − 0.515, t(N-2) = − 2.403, *P* = 0.029). There was also a positive correlation between hippocampal *Casp-3* expression and mean repeated training swimming time (*r*_*s*_ = 0.505, t(N-2) = − 2.265, *P* = 0.039). In the cerebellum and prefrontal cortex, Casp-3 gene expression negatively correlated with similar behavioral indicators (*r*_*s*_ = − 0.484, t(N-2) = − 2.212, *P* = 0.042) and (*r*_*s*_ = − 0.476, t(N-2) = − 2.164, *P* = 0.046), respectively.

Additionally, post-Ro25-6981 treatment protocol day-dependent correlation analyses revealed a negative correlation between hippocampal *Ascl1* expression and the mean time of repeated training on the 14th post-treatment day (*r*_*s*_ = − 0.714, t(N-2) = − 2.500, *P* = 0.047). Furthermore, on the 28th post-treatment day, there was a negative correlation between cerebellar Casp-3 expression and speed of reversal learning (*r*_*s*_ = − 0.750, t(N-2) = − 3.000, *P* = 0.019).

#### Summary of Gene Transcription Findings

**Fig. 10:**
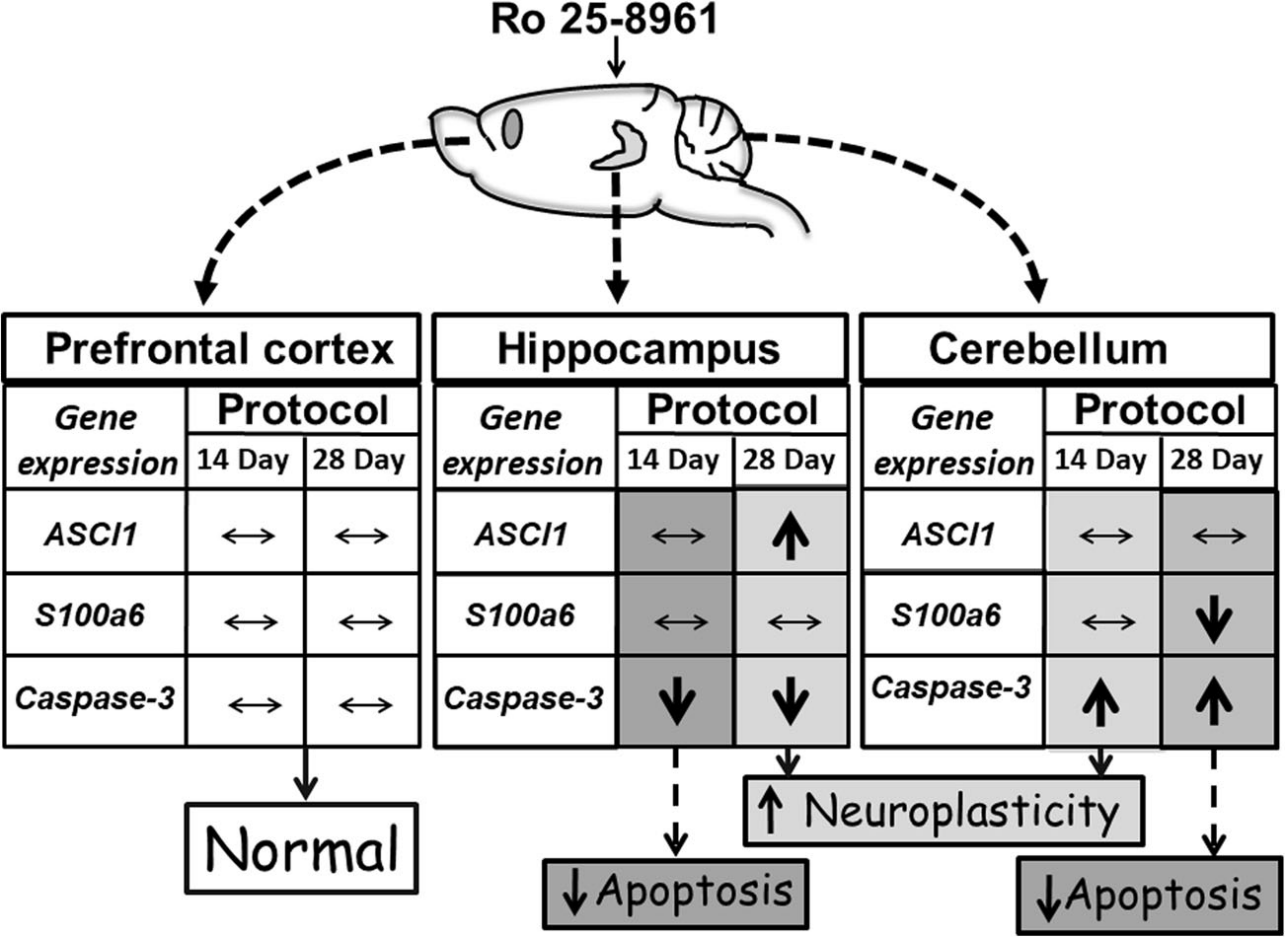
Summary tabulated scheme showing the overall influence of Ro25-6981 (5.0 mg/kg i.p.) 14 and 28 days after treatment on *AScl1*, *S100a6*, and *Casp-3* gene expression in trained rat prefrontal cortex, hippocampus, and cerebellum compared with controls. Horizontal double-headed arrows = no significant change, upward pointing arrows = significant increase, and downward pointing arrows = significant decrease

There is a very complex pattern of results with respect to the expression of genes in the study so the outcomes for *Ascl1*, *S100a6*, and *Casp-3* transcription in the three brain regions taken from the animal group which were retrained and treated with Ro25-6981 are presented in a more summarized form in Fig. 10. Hence, the notable outcomes were that hippocampal *Ascl1* expression was increased in the animals 28 days after Ro25-689 treatment, cerebellar *S100a6* expression was decreased 28 days after Ro25-6981, and hippocampal *Casp-3* expression was decreased both at 14 and 28 days after Ro25-6981 administration. In contrast, cerebellar *Casp-3* expression was increased at 14 and 28 days after dosing with Ro25-6981. There were no significant changes in the gene expressions in the prefrontal cortex (Fig. 10).

## Discussion

Multi-staged neurogenesis is important to learning processes (Hu et al. 2009). It is controlled by NMDA receptors involving different pathways initiating proliferation through CaMKIV phosphorylation and subsequent activation of CREB in precursor cells and differentiating granule neurons (Nácher and McEwen 2006, Nácher et al. 2007; Li et al. 2011; Bekiari et al. 2015). Correspondingly, findings concerning the role of glutamate receptors in spatial memory mechanisms support the hypothesis that their blockade may contribute towards stimulation of the critical stages of neurogenesis (Nácher and McEwen 2006; Hu et al. 2008).

Neurogenesis necessitates cellular progression from proliferation to maturation over a period up to 30 days (Aasebø et al. 2011). Thus, in the current study, this cellular developmental 30-day epoch was selected to investigate the possible involvement of different aged neuronal populations contributing to processes of spatial navigation.

In this context, there has been a special focus upon the NR2B subunit of NMDA receptors, antagonism of which actuates neurogenesis (Hu et al. 2008). In view of this, highly selective antagonism at receptors carrying this subunit, Ro25-6981 invokes a cascade of molecular events which involve CREB activation (Li et al. 2011) followed by remodeling of transcription regulation (Brown et al. 2011). Such outcomes may well lead to functional activation of neurogenetic processes which inevitably impact memory phenomena (Snyder et al. 2005; Costa et al. 2015).

The current data provides evidence that the qualitative influence of Ro25-6981 on spatial memory performance is very much a time-dependent entity over a period up to 28 days. Accordingly, we found that during repeated training with a fixed platform location 9–12 days (i.e., protocol (i) days 15–18, Fig. 1) after commencement of Ro25-6981 administration, animal learning capability was not significantly modified in comparison with controls. Likewise, injection of Ro25-6981 has been reported to have no effect on latency or path length to a MWM platform over a 5-day training period after drug treatment in mice (Duffy et al. 2008). However, in our study, 25–26 days (i.e., protocol days 31–32) after Ro25-6981 initial double dosing, there was an enhancement of platform performance in the repeated learning session (Fig. 2). The differences in latency to reach the platform reflected differences in travel distance to the platform (Fig. 3) but not the speed which was not significantly different between groups. This data may be associated with survival of newborn mature neurons which were integrated into existing neuronal nets to participate in processes underlying long-term visuospatial memory. However, subsequent reversal learning was not established on the 28th day (i.e., protocol (ii) day 33) after Ro25-6981 exposure. It may be postulated that the repeated learning procedure at the stage of reconsolidation of memory is characterized by activation of learning speed. These findings concur with data that administration of Ro25-6981 (10 mg/kg) selectively impairs the early phase of reversal learning. In view of this, spatial reversal learning but not set-shifting has been shown to be sensitive to Ro-25-6981 treatment (Clark et al. 2017). Additionally, it is of note from our findings that blockade of NR2B/NMDA receptors subsequently discloses a stimulant effect on memory reconsolidation at the repeated training stage of the experimental protocol where stored memories become more labile. On this matter, the converse has been demonstrated that blockade of reconsolidation results in permanent amnesia (Nader and Hardt 2009; Lee 2009). Furthermore, recent data has suggested that molecular mechanisms which are prompted during the initial stage of recall actually operate to restrict the early extinction of memory (Trent et al. 2015).

Gene expression of protein participants (Casp-3, Ascl1, and S100a6) involved in regulation of the proliferation and differentiation stages of neurogenesis has been previously implicated in visuospatial memory formation (Gruden et al. 2013). The present findings signify complex chronological and interstructurally linked brain gene expression associations with hippocampal-dependent memory. Thus, in the hippocampus, prefrontal cortex, and cerebellum, 14 days after initial Ro25-6981 injection, the expression of *S100a6* and *Ascl1* in trained animals did not differ from controls. Therefore, it may be postulated that blockade of NR2B/NMDA receptors is not crucial to modification of the proliferation and differentiation stages of neural development but may be important to the survival of differentiated neurons. This notion is supported by our data concerning the constrained expression of *Casp-3* in the hippocampus (14-day post-treatment) where a decrease in neuronal apoptosis is reciprocated by newborn cell survival and/or increased hippocampal plastic remodeling (Bailey et al. 2015). The opposite effect regarding *Casp-3* expression was found in the cerebellum which is involved not only in motor behavior (Swinny et al. 2005) but also spatial memory formation (Reeber et al. 2013). Hence, a significant increase in *Casp-3* expression in this brain area (Figs. 8, 9, and 10) might be correlated predominantly with motor circuitry. Recently however, it has been disclosed that blockade of NMDA receptors represses the glutamatergic contribution to cellular differentiation and growth needed for newborn GABA neuronal survival resulting in a shift towards apoptosis (Roux et al. 2015). Twenty-eight days post-Ro25-6981 treatment, a hippocampal elevation of *Ascl1* expression was observed, and this was associated with learning enhancement during repeated training. It is noteworthy that 28 days after Ro25-6981 dosing, an increased neuronal differentiation from elevated *Ascl1* gene activity in the hippocampus contrasted with inhibition of proliferation and *S100a6*-associated repeated training gene expression in the cerebellum. Interestingly, *Casp-3* expression, which is likely to be linked with programmed cell death and neural plasticity, was inhibited in the hippocampus but also markedly elevated in the cerebellum during the repeated training procedure 28 days after drug administration (Figs. 8, 9, and 10).

Arising from the gene expression/behavior correlation analyses, it was deduced that prefrontal cortical *Ascl1* expression is linked with individual capacities for learning which are not dependent on Ro25-6981 effect chronology in the current protocols. Hippocampal *Ascl1* and *Casp-3* expressions appear to be implicated in the early stages of memory formation during repeated training. This contrasts with cerebellar *Casp-3* expression, which is crucial to later stages along with increased repeated training or reversal learning speed (i.e., elements of neural plasticity which are connected with a strategy to find the platform). It was also concluded that increased prefrontal cortical *Ascl1* and *Casp-3* expression along with cerebellar *Casp-3* accompany rapid learning during repeated training. However, high levels of hippocampal *Casp-3* expression may be associated with slow learning during repeated training.

Clinical aspects of morbidity connected with human spatial memory have directed attention to higher mammals. It has been documented that chimpanzees have larger hippocampal and cerebellar volumes relative to bonobo apes (Hopkins et al. 2009). This may be related to a wider home range (≤ 560 km^2^) in chimpanzees (Shefferly 2005) likely to impose a bigger demand on spatial memory (Hassabis et al. 2009) versus the bonobos smaller home range (≤ 60 km^2^) (Hopkins et al. 2009; Fruth et al. 2016). These observations are accompanied by an expansion of knowledge regarding adult neurogenesis in the hippocampus (Kempermann 2012) and cerebellum (Gruden et al. 2013) though this developmental component is not well established in humans (Bergmann et al. 2015). Selective blockade and/or upregulation of NR2B/NMDA receptors gives rise to improved memory retrieval aptitude through gene regulation of basic cell birth and death processes, and involvement of new mature neurons. These findings are therefore manifestly incentive towards future diagnosis and treatment strategies for clinical cognitive deficits.

## Conclusions

In summary, Ro25-6981 blockade of NR2B/NMDA receptors enhanced spatial memory retrieval in rats but did not influence visuospatial reversal learning. It also modified brain *S100a6*, *Ascl1*, and *Casp-3* cell regulator gene expression and in all probability actuated neurogenesis possibly implicating new mature neurons in memory retrieval.
